# Conductivity and aging behavior of Sr(Ti_0.6_Fe_0.4_)_1−*x*_O_3−*δ*_ mixed conductor materials

**DOI:** 10.1039/d3ra00583f

**Published:** 2023-03-15

**Authors:** Ke Shan, Davoud Dastan, Zhong-Zhou Yi, Mustafa K. A. Mohammed, Xi-Tao Yin, Abdelmajid Timoumi, Alex S. Weidenbach

**Affiliations:** a School of Chemistry and Resource Engineering, Honghe University Yunnan Province 661199 China yizhongzhou@126.com; b Department of Materials Science and Engineering, Cornell University Ithaca NY 14850 USA d.dastan61@yahoo.com; c University of Warith Al-Anbiyaa 56001 Karbala Iraq mustafa_kareem97@yahoo.com; d School of Physics and Optoelectronic Engineering, Ludong University Yantai Shandong Province 264000 China; e Department of Physics, Faculty of Applied Science, Umm AL-Qura University P. O. Box 715 Makkah Saudi Arabia; f School of Electrical and Computer Engineering, Georgia Institute of Technology Atlanta GA 30332 USA

## Abstract

Perovskite materials play a significant role in oxygen sensors due to their fascinating electrical and ionic conductivities. The sol–gel technique was employed to prepare various compositions of B-site-deficient Fe-doped SrTiO_3_ (iron-doped strontium titanate) or Sr(Ti_0.6_Fe_0.4_)_1−*x*_O_3−*δ*_, where *x* = 0.01, 0.02, and 0.03. The XRD results revealed that the principle crystalline phase of the samples was the cubic perovskite structure. The B-site deficiency improved the ionic and total conductivities of Sr(Ti_0.6_Fe_0.4_)_1−*x*_O_3−*δ*_. A small polaron conduction behavior occurred in the total electrical conductivity. The XPS results showed that the oxygen vacancy value decreased with the rise in the amount of B-site deficiencies. A lower B-site deficiency amount could produce more oxygen vacancies in the lattice but resulted in the ordering of vacancies and then lower ionic conductivity. The aging behavior was caused by the ordering of oxygen vacancies and resulted in a degeneration of electrical features under a long service time. Conversely, augmentation of the B-site deficiency amount inhibited the tendency for the ordering of oxygen vacancies and then promoted the electrical performance under a long usage time. The conduction mechanism of oxygen ions through oxygen vacancies was thoroughly investigated and discussed. The current study presents a feasible approach to ameliorate the physical features of conductors through doping the B-site of the perovskite layer with Fe, which would be a fruitful approach for numerous applications, including oxygen sensors and fuel cells anodes.

## Abstract 2

Sol–gel technique is employed to prepare various compositions of B-site-deficient Fe-doped SrTiO_3_ (iron-doped strontium titanate) or Sr(Ti_0.6_Fe_0.4_)_1−*x*_O_3−*δ*_, where *x* = 0.01, 0.02, and 0.03.

## Graphical abstract

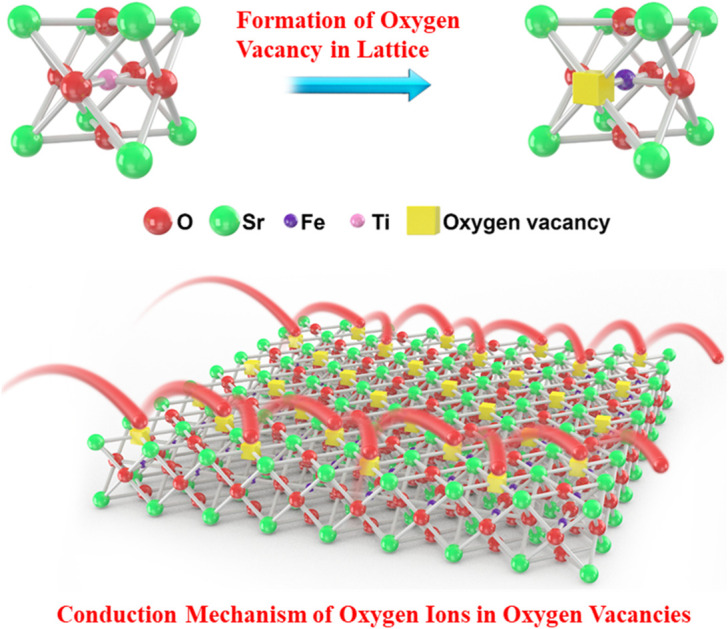

## 1. Introduction

Perovskite-type oxides are promising candidates for fuel cell anodes, sensors, and other membrane devices.^1–7^ Among the oxides, the perovskite oxides with a cubic structure (ABO_3_) are one of the most promising applications due to their ability to tolerate a high level of dopant ions and open migration paths of oxygen ions.^8–11^ Many significant attempts have been made to promote both the ionic and electronic conductivity of oxygen sensors, oxygen membrane devices, anodes of solid oxide fuel cells and so on owing to their adjustable electrical properties and good chemical stability.^12–23^

In order to promote combined ionic–electronic conductivity for oxygen sensors, several significant attempts have been made. Among the cubic perovskite oxides, strontium titanate is a chemically stable perovskite material under oxidizing and reducing atmospheres. However, the pure strontium titanate material has low electronic and ionic conductivities, which prevents its practical use for sensors and as electrodes of solid oxide fuel cells (SOFCs) and other devices.^24–26^

According to many researchers, the electrical conductivity could be improved or influenced by donor doping, such as La(iii) or Y(iii) ions on the Sr-site or Nb(v) on the Ti-site.^27–31^ Similarly, the ionic conductivity could be enhanced by acceptor doping on the Ti-site, such as Cr^3+^, Fe^3+^, Ni^2+^, and Sc^3+^.^32–37^ It has been reported that donor or acceptor doping in SrTiO_3_ can promote both ionic and electronic conductivity, respectively, and the two kinds of conduction behavior are generally distinct.^38^ Nonetheless, according to our prior study, A- or B-site loading can have a substantial impact on the ionic and electrical conductivity.^39–42^ The impact of A- and/or B-site loading on both the ionic and electronic conductivity, however, cannot be used to infer the dependent effect of the separate loading on the electronic and ionic conductivity of oxygen sensors.

In other words, there are a few possibilities: (1) A- or B-site doping may promote the electronic and ionic conductivities simultaneously; (2) A- or B-site doping could be unfavorable to the enhancement of the electronic and ionic conductivities simultaneously; or (3) A- or B-site doping may promote the electronic (or ionic) conductivity but result in the decrease in the ionic (or electronic) conductivity. In addition, the effect of A- and/or B-site deficiencies in the perovskite structure on the electronic as well as ionic conductivity can be evident due to the fact that the deficiency can define the level and kinds of imperfections. For example, A-site deficiency can lead to a decrease in the electron content, which can cause a lower electronic conductivity. Similarly, B-site deficiency can result in a decrease of the amount of oxygen vacancies, which can cause a lower ionic conductivity.

However, we found that the influence of the deficiency on the electrical conductivity was not limited to this. Therefore, we tried to find the relation between the produced defects amount as a deficiency and the electrical conductivity. In this paper, nonstoichiometric Fe-doped SrTiO_3_ mixed conductor materials were synthesized by a sol–gel method. The structural characteristics, microstructure, total conductivity, and ionic conductivity of Sr(Ti_0.6_Fe_0.4_)_1−*x*_O_3−*δ*_ (with the ‘*x*’ values of 0.01, 0.02, and 0.03) were evaluated. Especially, the effect of the B-site nonstoichiometric amount on the concentration of oxygen vacancies was examined by XPS analysis. The aging behavior of materials is significantly important because it changes the electrical properties under a long usage time and certain temperature. Therefore, the aging behavior was discussed by the ordering of the oxygen vacancies in the lattice.

## 2. Experimental details

Iron-doped strontium titanate with B-site deficiencies [Sr(Ti_0.6_Fe_0.4_)_1−*x*_O_3−*δ*_ (with the ‘*x*’ values of 0.01, 0.02, and 0.03)] were synthesized by a sol–gel route from Sr(CH_3_COO)_2_·2H_2_O (AR), Ti(CH_3_CH_2_CH_2_CH_2_O)_4_ (AR), and Fe_2_O_3_ (AR).^43–47^ Here, strontium acetate interplayed with butyl titanate to develop the sol and concurrently Fe_2_O_3_ dispersed homogeneously on the sol during moderate magnetic stirring. The product was obtained in the form of a powder *via* sol-drying at the temperature of 50 °C. The dry gelatin powder was ground using agate mortar pestle and subsequently annealed at 1100 °C for 10 h under ambient conditions. The annealed powder was compressed in a disc form (8 mm diameter and ∼2 mm thickness) in the mold by uniaxial pressing (*ca.* under 50 MPa). The green tablets were annealed at a temperature of 1350 °C for 5 h at room temperature to get dense specimens for further analysis, including by XRD, SEM, XPS, and electrical property measurements.

The phase composition was achieved by XRD (X-ray diffraction, Rigaku D/max-A X-ray diffractometry, and Cu Kα radiation). Scanning electron microscopy (SEM, HITACHI-SU8010) was used to evaluate the microstructures of the annealed specimens. The oxidation states of the existing elements within the samples were measured *via* X-ray photoelectron spectroscopy (XPS, Thermo Scientific) with Al Kα. The total electrical conductivity was tested by electrochemical impedance spectroscopy (EIS) between 400 °C and 950 °C at room temperature. The oxygen ionic conductivity was investigated by the electron-blocking electrode approach in the temperature range of 600 °C to 1000 °C under ambient conditions. The electrodes for the electrical property measurements were assembled by coating Pt paste on both sides of sintered tablets and further baked at 1100 °C for 1 h. The electrode setup for the ionic conductivity test has already been explained thoroughly elsewhere.^39^ All the analyses were carried out under the equilibrium of each temperature point. The mixed conductivity measurements (total and ionic conductivities) were performed for every 50 °C. The aging behavior was measured at 800 °C for 24 h and the measurement was taken in intervals of 2 h.

## 3. Results and discussion

### 3.1. XRD analysis

**Fig. 1:**
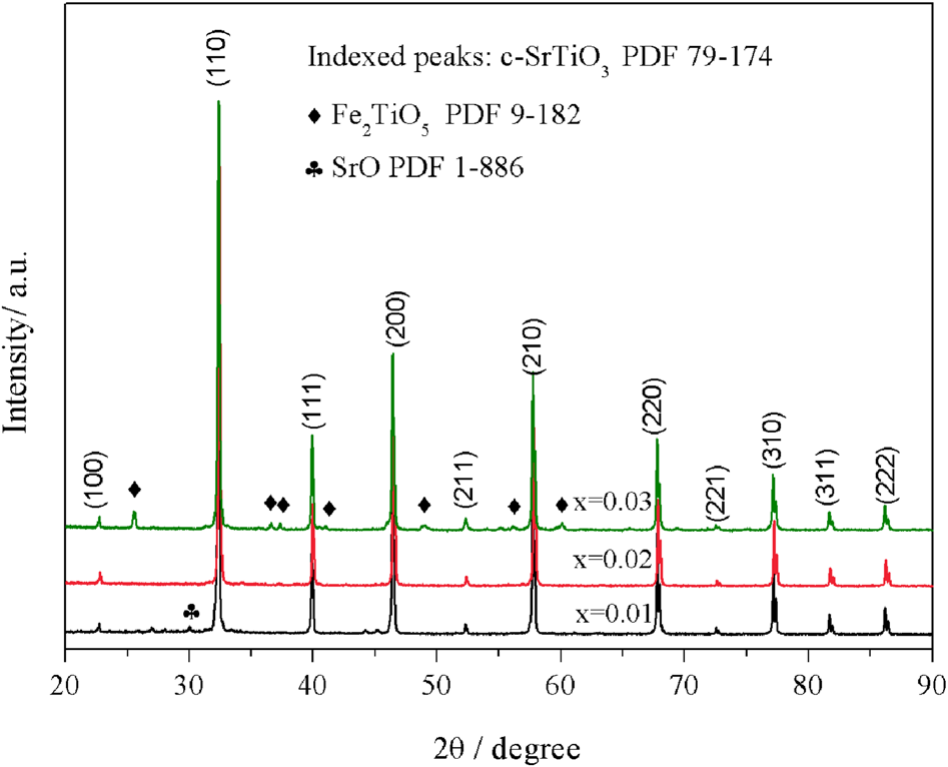
XRD patterns of Sr(Ti_0.6_Fe_0.4_)_1−*x*_O_3−*δ*_ (for the values of *x* = 0.01, 0.02, and 0.03) after firing at 1350 °C for 5 h in the air.

Sr(Ti_0.6_Fe_0.4_)_1−*x*_O_3−*δ*_ (with the ‘*x*’ values of 0.01, 0.02, and 0.03) upon being sintered at 1350 °C for 5 h in an air atmosphere was evaluated *via* XRD for determining the phase composition. The results are shown in Fig. 1. The referenced diffraction peaks corresponded to those of cubic SrTiO_3_ in PDF 79-174 and were part of the cubic perovskite structure (*Pm*3̄*m*). The peaks at 25.57°, 36.59°, 37.42°, 41.14°, 56.33°, and 60.05° corresponded to those of Fe_2_TiO_5_ in PDF 9-182 for Sr(Ti_0.6_Fe_0.4_)_0.97_O_3−*δ*_ and the peaks at 30.00° and 30.98° were consistent with that of SrO in PDF 1-886 for Sr(Ti_0.6_Fe_0.4_)_0.99_O_3−*δ*_. Theoretically, the ionic radius of Fe^3+^ (0.0645 nm for six connections) is equivalent to that of Ti^4+^ (0.0605 nm for six connections),^40^ therefore Fe^3+^ should dissolve into the perovskite lattice to form the substitutional solid solution. However, the introduction of B-site nonstoichiometry leads to the formation of other phases for Sr(Ti_0.6_Fe_0.4_)_0.97_O_3−*δ*_ and Sr(Ti_0.6_Fe_0.4_)_0.99_O_3−*δ*_. For further exploration of the influence of B-site deficiencies on the perovskite structure, the tolerance factor (*t*) should be calculated because the tolerance factor can directly indicate the reason for the structural change. The tolerance factor can be calculated by the average radii of the A-site (*r*_A_), B-site (*r*_B_) cations, and oxygen ions (*r*_O_), as shown in eqn (1), indicating that the cubic perovskite can exist in the range of 0.95 < *t* < 1.04.^41^1
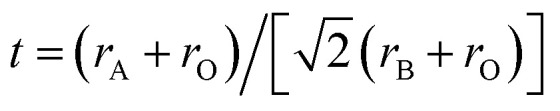


The calculated tolerance factors of Sr(Ti_0.6_Fe_0.4_)_1−*x*_O_3−*δ*_ with the ‘*x*’ values of 0.01, 0.02, and 0.03 were 0.9967, 0.9998, and 1.0029, respectively, indicating that B-site deficiency did not affect the formation of the principal crystalline phase of the cubic perovskite structure, and showing that the crystal structure of *x* = 0.02 was closest to the ideal cubic perovskite structure (*t* = 1). Therefore, as can be seen from Fig. 1, there was no second phase for Sr(Ti_0.6_Fe_0.4_)_0.98_O_3−*δ*_. Rietveld refinement of the obtained XRD spectra was used to address the influence of the B-site deficiencies on the specimens' lattice volume and parameter.

**Fig. 2:**
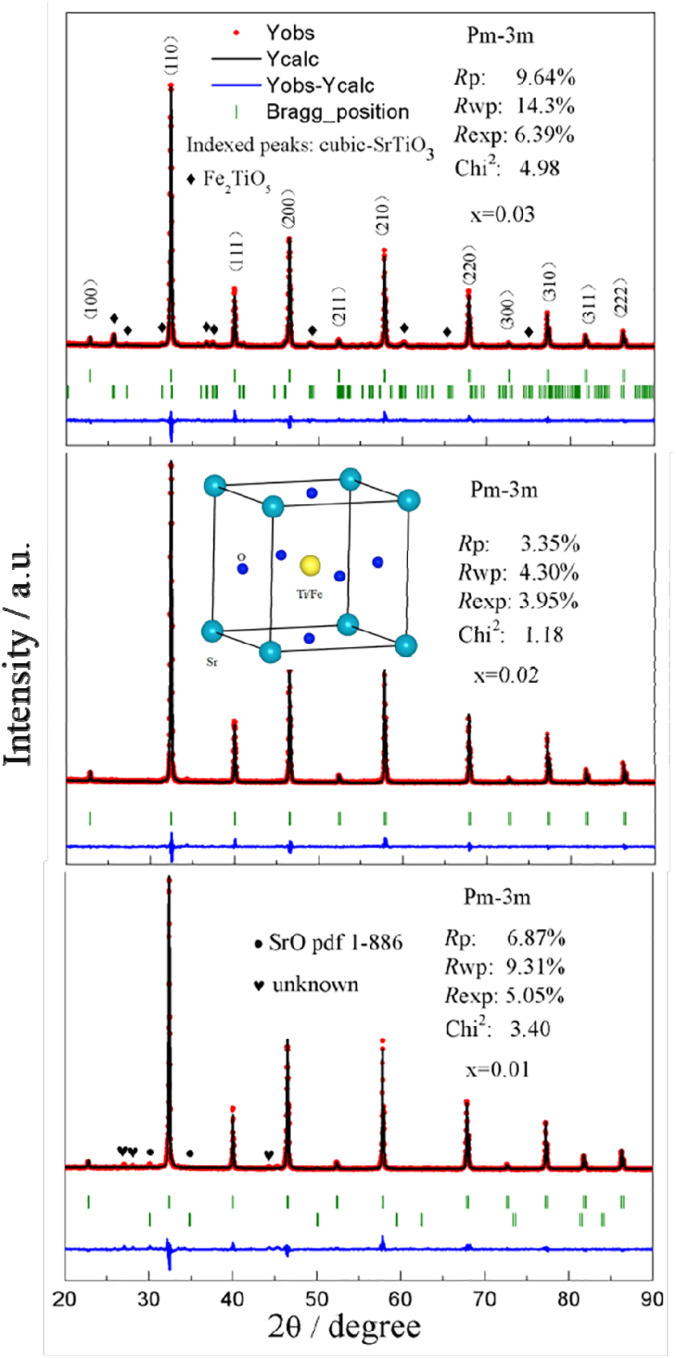
Rietveld refinement in Sr(Ti_0.6_Fe_0.4_)_1−*x*_O_3−*δ*_ (for the values of *x* = 0.01, 0.02, and 0.03).

Fig. 2 shows that the lattice parameters were 3.9037, 3.9029, and 3.9042 Å and the lattice volumes were 59.486, 59.451, and 59.520 Å^3^ for the various values of *x* = 0.01, 0.02, and 0.03, accordingly. Due to the formation of Fe_2_TiO_5_ and SrO for Sr(Ti_0.6_Fe_0.4_)_0.97_O_3−*δ*_ and Sr(Ti_0.6_Fe_0.4_)_0.99_O_3−*δ*_, respectively, the lattice parameter and lattice volume of Sr(Ti_0.6_Fe_0.4_)_0.97_O_3−*δ*_ and Sr(Ti_0.6_Fe_0.4_)_0.99_O_3−*δ*_ were greater than those for Sr(Ti_0.6_Fe_0.4_)_0.98_O_3−*δ*_. Therefore, according to the results of the tolerance factor and Rietveld refinement, the lattice distortion of Sr(Ti_0.6_Fe_0.4_)_0.98_O_3−*δ*_ was negligible.

### 3.2. SEM analysis

**Fig. 3:**
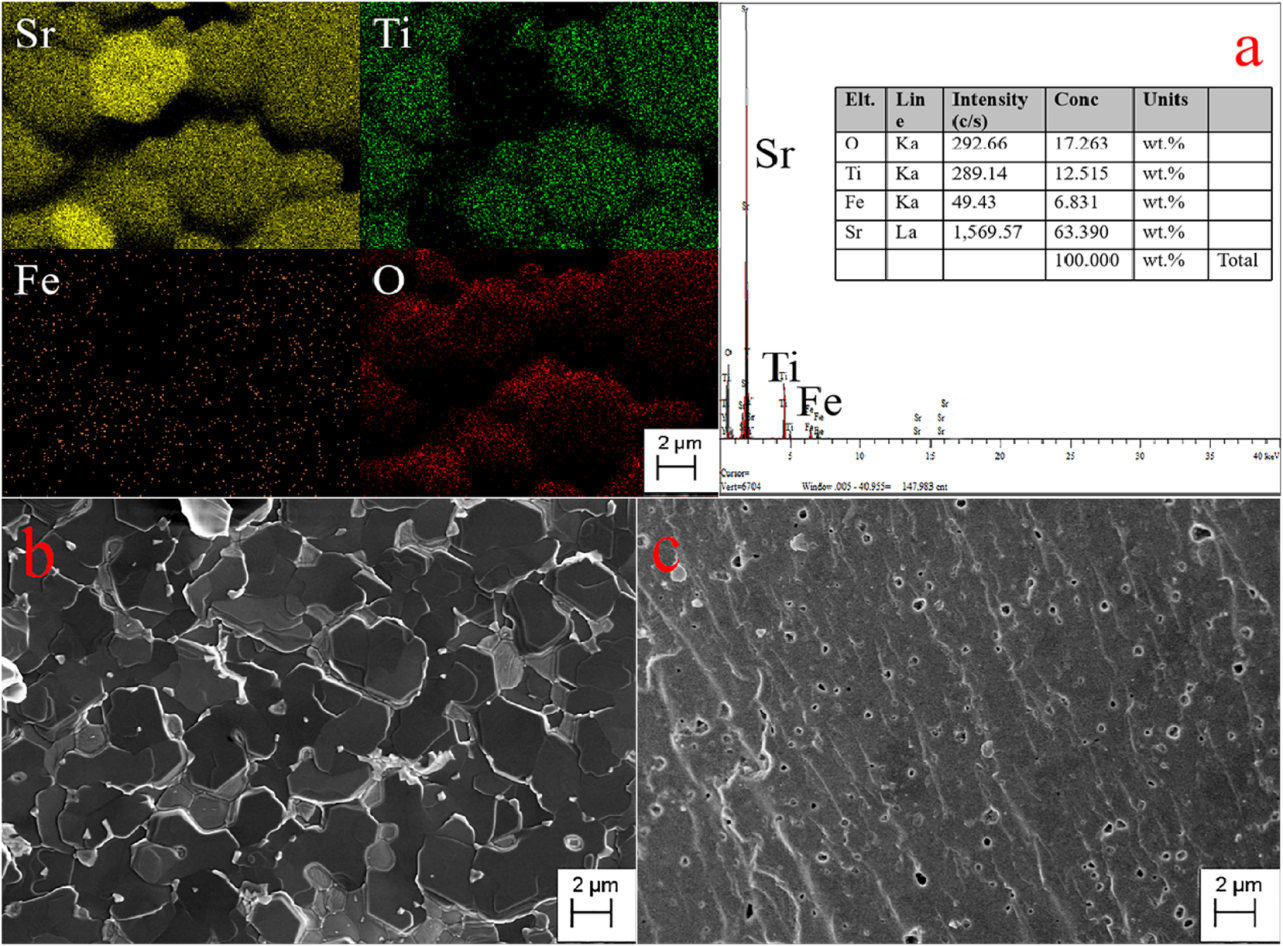
Elemental distribution maps and EDS of the fracture surface of Sr(Ti_0.6_Fe_0.4_)_0.97_O_3−δ_ (a) and SEM micrographs of the fracture surfaces of Sr(Ti_0.6_Fe_0.4_)_1−*x*_O_3−*δ*_: (b) *x* = 0.01 and (c) *x* = 0.03.

The SEM, EDS images, and elemental distribution mappings of the fractured surface of the Sr(Ti_0.6_Fe_0.4_)_0.97_O_3−*δ*_ specimen are exhibited in Fig. 3. As shown from the mappings, the proportion of Sr, Fe, O, and Ti elements is homogeneous. According to the mapping results, the Fe element can be dissolved into the SrTiO_3_ structure. The SEM micrographs of the fracture surfaces of the Sr(Ti_0.6_Fe_0.4_)_1−*x*_O_3−*δ*_ samples (with the ‘*x*’ values of 0.01 and 0.03) are shown in Fig. 3b and c. It could be seen that the porosity increased and grain size decreased obviously with the augmentation in the B-site deficiency, indicating that the rise in B-site deficiencies may be a disadvantage for the increased density of the Fe-loaded SrTiO_3_ composites.

### 3.3. Mixed electronic–ionic conductivity analysis and aging behavior

**Fig. 4:**
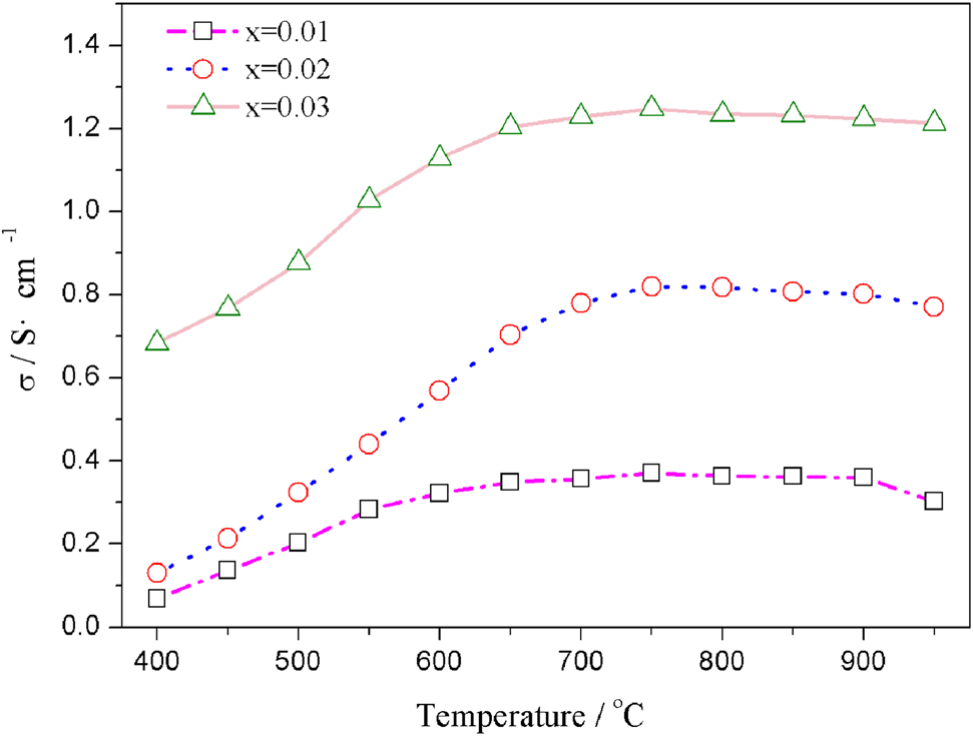
Temperature dependences of the total electrical conductivity of Sr(Ti_0.6_Fe_0.4_)_1−*x*_O_3−*δ*_ (for the values of *x* = 0.01, 0.02, and 0.03).

The relation curves of the electrical conductivity of Sr(Ti_0.6_Fe_0.4_)_1−*x*_O_3−*δ*_ samples with different nonstoichiometries and temperatures are presented in Fig. 4. Upon increasing the B-site deficiency amount, the total conductivity of Sr(Ti_0.6_Fe_0.4_)_1−*x*_O_3−*δ*_ (with ‘*x*’ values of 0.01, 0.02, and 0.03) clearly increased. In addition, the electrical conductivity of the Sr(Ti_0.6_Fe_0.4_)_1−*x*_O_3−*δ*_ samples with different nonstoichiometries increased through to a maximum at 750 °C with the temperature increasing, and then decreased slightly, which demonstrated a small polaron conduction behavior of the Sr(Ti_0.6_Fe_0.4_)_1−*x*_O_3−*δ*_ materials. A small polaron is made up of charge carriers and lattice deformation originating from the electrons or holes.^42,48–50^ The conductivity can be determined from the carrier concentration and carrier migration rate.

**Fig. 5:**
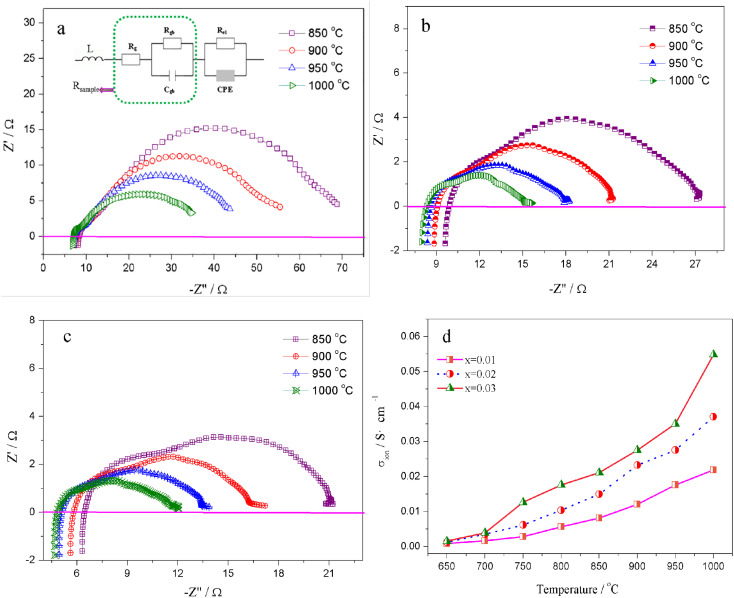
Nyquist graphs of (a) Sr(Ti_0.6_Fe_0.4_)_0.99_O_3−*δ*_, (b) Sr(Ti_0.6_Fe_0.4_)_0.98_O_3−*δ*_, and (c) Sr(Ti_0.6_Fe_0.4_)_0.97_O_3−*δ*_ for the ionic conductivity at various temperatures, and (d) temperature dependence of the ionic conductivity of Sr(Ti_0.6_Fe_0.4_)_1−*x*_O_3−*δ*_ (for the values of *x* = 0.01, 0.02, and 0.03).

The migration of tiny polarons is equivalent to negative charge carriers (electrons) at low temperatures because of the weak electron–lattice distortion coupling, therefore a rise in the operating temperature (≤850 °C) resulted in an augmentation in the electrical conductivity. Although, at higher temperatures, tiny polaron hopping induced by the enhanced electron–lattice deformation recombination resulted in low mobility, so the electrical conductivity decreased with the temperature increasing (>850 °C). The Nyquist plots for the ionic conductivity of Sr(Ti_0.6_Fe_0.4_)_1−*x*_O_3−*δ*_ (with the ‘*x*’ values of 0.01, 0.02, and 0.03) *versus* temperature are delineated in Fig. 5a–c. As could be evidently seen, the resistances of the grain (*R*_g_) and grain boundary (*R*_gb_) diminished after increasing the applied temperature, and these phenomena were led by the decreased intercept and semicircle in the plots, according to Fig. 5a–c. Fig. 5d shows the relation between the ionic conductivity of Sr(Ti_0.6_Fe_0.4_)_1−*x*_O_3−*δ*_ (with the ‘*x*’ values of 0.01, 0.02, and 0.03) and the applied temperature. The ionic conductivity rose gradually as the applied temperature and B-site deficiencies increased, indicating that the B-site deficiencies possibly promoted the concentration of oxygen vacancies as well as the movement rate of oxygen ions. The imperfections in the Fe-loaded SrTiO_3_ lattice can be expressed by the following eqn (2) based on the concept of defects in chemistry:2
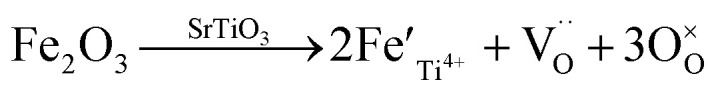


**Fig. 6:**
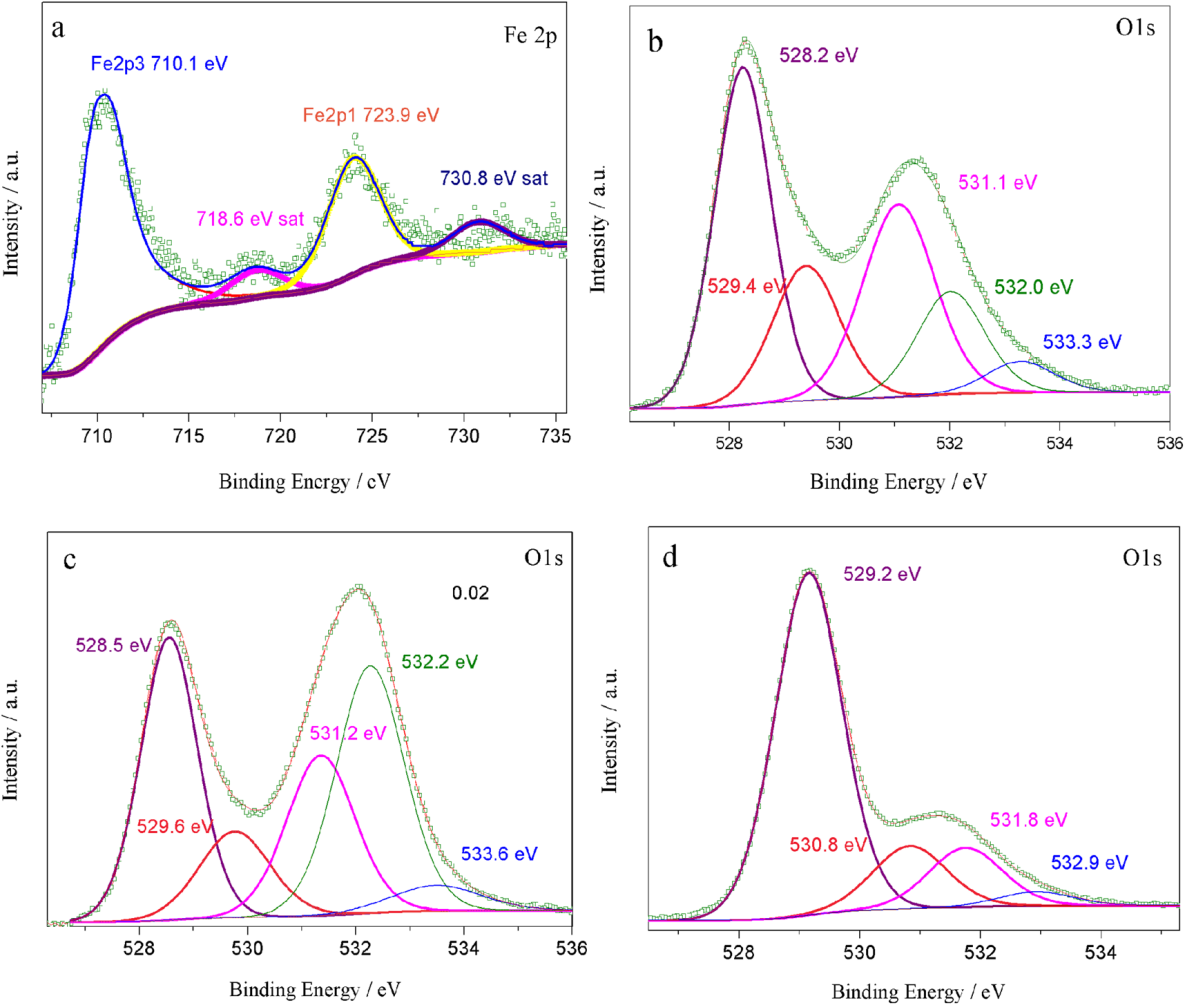
XPS spectra of Sr(Ti_0.6_Fe_0.4_)_1−*x*_O_3−*δ*_: (a) Fe 2p, (b–d) O 1s for the values of *x* = 0.01, 0.02, and 0.03, accordingly.

According to eqn (2), an increase in B-site deficiencies should result in a decrease in oxygen vacancies content 
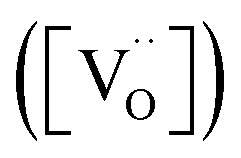
. However, the experimental results showed that B-site deficiencies promoted the ionic conductivity. To better explain the impact of B-site deficiencies on the ionic conduction, it was necessary to carry out XPS measurements. Fig. 6a shows the Fe 2p XPS spectrum of Sr(Ti_0.6_Fe_0.4_)_1−*x*_O_3−*δ*_. The observed binding energies of the Fe 2p1 and Fe 2p3 peaks at 723.9 and 710.1 eV corresponded to Fe^3+^, indicating that the valence state of the doped Fe ions was +3. Therefore, oxygen vacancies were introduced in the lattice by Fe-doping. The concentrations of oxygen vacancies in the Fe-replaced samples were calculated *via* the atomic content proportion obtained from the O 1s XPS spectra (Fig. 6b–d).^42^

The vacancy contents were calculated are 0.49, 0.48, and 0.16 (Table 1). Thus we could determine the molecular formulas of Fe-doped SrTiO_3_ as Sr(Ti_0.6_Fe_0.4_)_0.99_O_2.51_, Sr(Ti_0.6_Fe_0.4_)_0.98_O_2.52_, and Sr(Ti_0.6_Fe_0.4_)_0.97_O_2.84_, which agreed with eqn (2). Commonly, a high level of oxygen vacancies can promote the ionic conductivity. Herein, B-site deficiencies led to a decrease in the amount of oxygen vacancies and an increase in the ionic conductivity. In this regard, an ordering of the oxygen vacancies for the cubic perovskite material should exist in the samples.^39^ The partial ordering of oxygen vacancies is probably a key factor determining the ionic conductivity of perovskites.

**Table 1:** Percentage oxygen vacancy concentration based on the atomic concentration percentage of O 1s (MB and LB represent the medium- and low-binding energy)

B-site deficiency content (*x*)	Atomic concentration percent of O 1s			MB/LB values
*x* = 0.01	37.39	17.81	27.13	49%
*x* = 0.02	29.66	12.06	20.04	48%
*x* = 0.03	69.49	14.10	13.29	16%

**Fig. 7:**
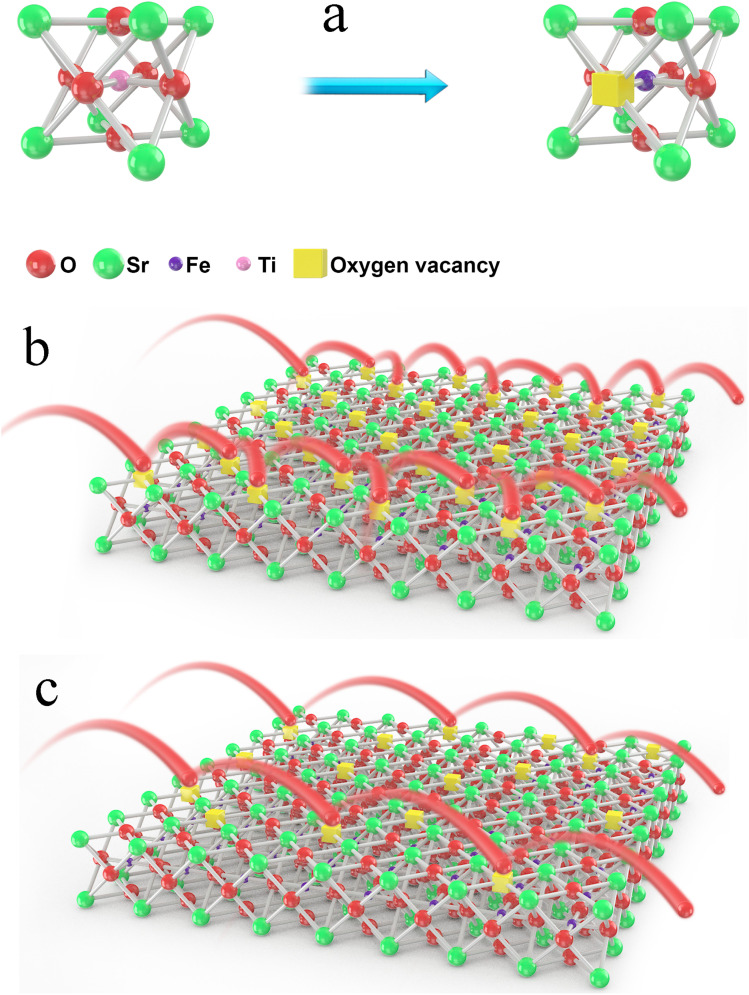
Formation of an oxygen vacancy in the lattice (a) and the different conduction mechanisms of oxygen ions in oxygen vacancies (b) and (c).

The formation of oxygen vacancies in the lattice and the different conduction mechanisms of oxygen ions in oxygen vacancies were thoroughly investigated, as demonstrated in Fig. 7. As can be clearly seen from Fig. 7b, too many oxygen vacancies can result in the formation of an ordered arrangement and then lead to cumbersome oxygen ion transition steps. On the contrary, an appropriate oxygen vacancy concentration can promote the oxygen ions jumping, as shown in Fig. 7c. In particular, the ordering may be similar to the activation energy. This may suggest that the majority of the oxygen vacancies contribute to the ordered microdomains and that the surrounding environment for the oxygen vacancies was almost the same.

The ordering of oxygen vacancies led to a decrease in the ionic conductivity. Therefore, when the oxygen vacancy concentration was within a certain range, it was accurate or even the rule that the ionic conductivity increased with the oxygen vacancy concentration increasing. Therefore, the oxygen vacancy concentration value was 0.16 for *x* = 0.03, and the ionic conductivity was the highest, but the ordering of oxygen vacancies, as shown in Fig. 7b, resulted in a decrease in the ionic conductivity for the other two samples. In other words, compared with a high B-site deficiency level, a lower B-site deficiency amount could produce more oxygen vacancies in the lattice but result in an ordering of the vacancies. To further verify the ordering of the oxygen vacancies, the aging behavior of the materials was measured.

**Fig. 8:**
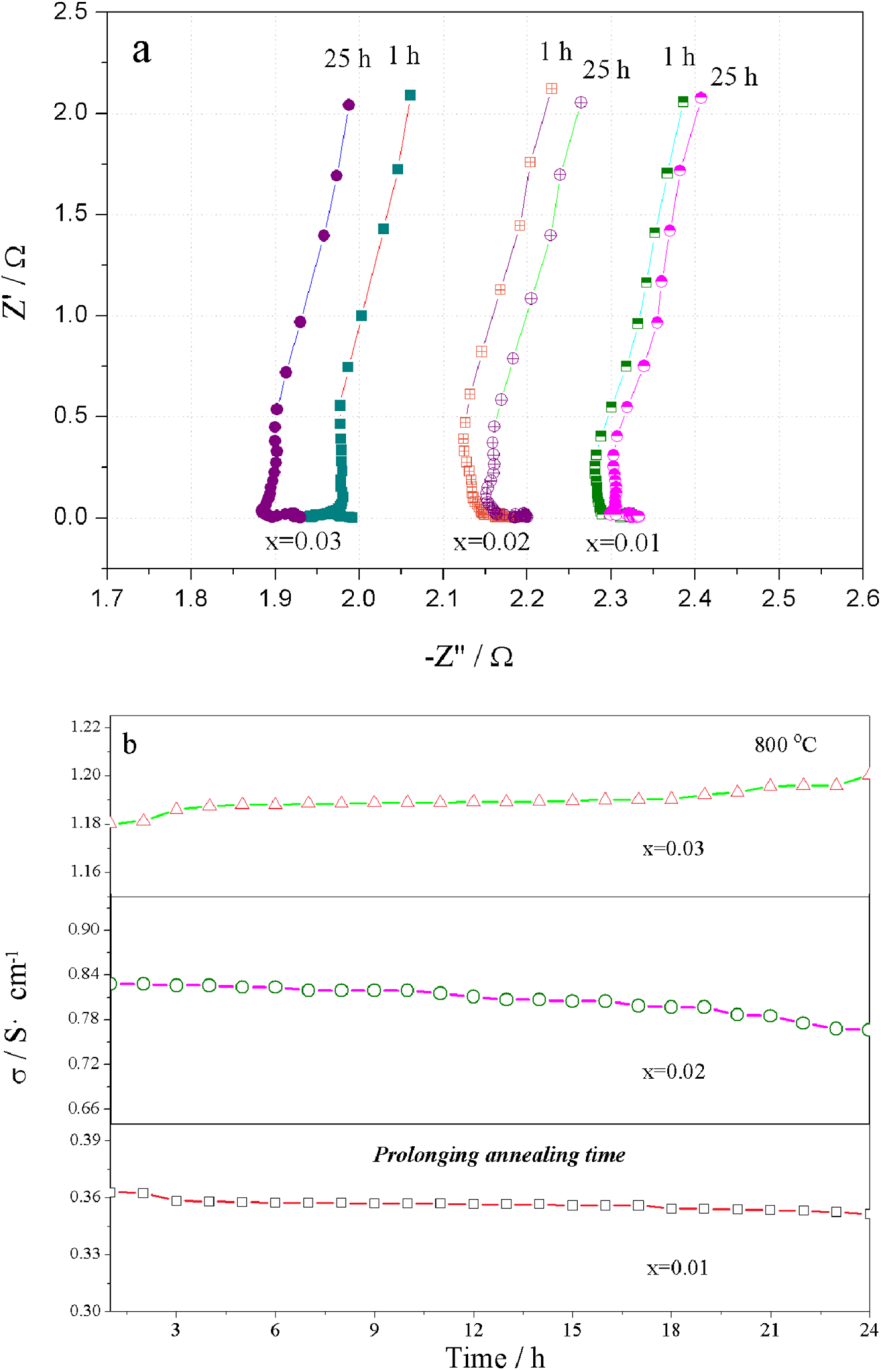
(a) Nyquist graphs of Sr(Ti_0.6_Fe_0.4_)_1−*x*_O_3−*δ*_ (for the values of *x* = 0.01, 0.02, and 0.03) as a function of time for the total electrical conductivity and (b) electrical conductivity curves of Sr(Ti_0.6_Fe_0.4_)_1−*x*_O_3−*δ*_ (for the values of *x* = 0.01, 0.02, and 0.03) calcinated at 800 °C for 24 h.

The Nyquist plots for the total electrical conductivity of Sr(Ti_0.6_Fe_0.4_)_1−*x*_O_3−*δ*_ (with the ‘*x*’ values of 0.01, 0.02, and 0.03) *versus* time are displayed in Fig. 8a. As can be clearly seen, the resistance increased for Sr(Ti_0.6_Fe_0.4_)_0.98_O_3−*δ*_ and Sr(Ti_0.6_Fe_0.4_)_0.99_O_3−*δ*_ and then decreased for Sr(Ti_0.6_Fe_0.4_)_0.97_O_3−*δ*_ upon prolonging the time. The aging behavior resulted from the ordering of the oxygen vacancies, which resulted in a decrease in the electrical conductivity over a long time and at a certain temperature. Fig. 8b shows the electrical conductivity isotherms of Sr(Ti_0.6_Fe_0.4_)_1−*x*_O_3−*δ*_ at 800 °C as a function of the annealing time. As shown in Fig. 8b, the electrical conductivity of Sr(Ti_0.6_Fe_0.4_)_0.99_O_3−*δ*_ and Sr(Ti_0.6_Fe_0.4_)_0.98_O_3−*δ*_ decreased gradually with the annealing time prolonging. This was different from the electrical conductivity of the two samples of Sr(Ti_0.6_Fe_0.4_)_0.97_O_3−*δ*_, which were found to increase significantly under a longer service time. By contrast, the aging behavior of Sr(Ti_0.6_Fe_0.4_)_0.97_O_3−*δ*_ caused an increasing conductivity value after 24 h. Thus, the increase in the B-site deficiency amount inhibited the tendency for oxygen vacancies ordering and thus promoted the electrical performance under a longer usage time.

## 4. Conclusion

Mixed conductors based on Fe-doped SrTiO_3_ or Sr(Ti_0.6_Fe_0.4_)_1−*x*_O_3−*δ*_, where *x* = 0.01, 0.02, 0.03, perovskite layers were successfully fabricated *via* a sol–gel technique. The principal crystalline phase of Sr(Ti_0.6_Fe_0.4_)_1-*x*_O_3−*δ*_ with ‘*x*’ values of 0.01, 0.02, 0.03 calcinated at 1350 °C for 5 h at room temperature belonged to the cubic perovskite. The total conductivity was enhanced with the increase in the amount of B-site deficiencies and it exhibited a small polaron conduction behavior. The ionic conductivity was also enhanced upon increasing the B-site deficiencies. Although, the rise in B-site deficiency amount could result in a lower concentration of oxygen vacancies, the low B-site deficiency level (high oxygen vacancy concentration) could enhance the ordering of oxygen vacancies in the cubic perovskite material. Such vacancy ordering would be responsible for a lower ionic conductivity. The increase in the B-site deficiency amount inhibited the tendency for oxygen vacancy ordering, thus promoting the electrical performance under a longer usage time. In this study, a simple approach was employed to improve the physical properties of conductors by doping the B-site of the perovskite layer with Fe, which would be advantageous for several applications, such as oxygen sensors and fuel cell anodes.

## Conflicts of interest

The authors declare that they have no known competing financial interests or personal relationships that could have appeared to influence the work reported in this paper.

## Supplementary Material
